# Serum suPAR and syndecan-4 levels predict severity of community-acquired pneumonia: a prospective, multi-centre study

**DOI:** 10.1186/s13054-018-1943-y

**Published:** 2018-01-24

**Authors:** Qiongzhen Luo, Pu Ning, Yali Zheng, Ying Shang, Bing Zhou, Zhancheng Gao

**Affiliations:** Department of Respiratory & Critical Care Medicine, Peking University People’s Hospital, Beijing, People’s Republic of China

**Keywords:** suPAR, Syndecan-4, Community acquired pneumonia, Severity, Mortality

## Abstract

**Background:**

Community-acquired pneumonia (CAP) is a major cause of death worldwide and occurs with variable severity. There are few studies focused on the expression of soluble urokinase-type plasminogen activator receptor (suPAR) and syndecan-4 in patients with CAP.

**Methods:**

A prospective, multi-centre study was conducted between January 2014 and December 2016. A total of 103 patients with severe CAP (SCAP), 149 patients with non-SCAP, and 30 healthy individuals were enrolled. Clinical data were recorded for all enrolled patients. Serum suPAR and syndecan-4 levels were determined by quantitative enzyme-linked immunosorbent assay. The t test and Mann–Whitney U test were used to compare between two groups; one-way analysis of variance and the Kruskal–Wallis test were used to compare multiple groups. Correlations were assessed using Pearson and Spearman tests. Area under the curve (AUCs), optimal threshold values, sensitivity, and specificity were calculated. Survival curves were constructed and compared by log-rank test. Regression analyses assessed the effect of multiple variables on 30-day survival.

**Results:**

suPAR levels increased in all patients with CAP, especially in severe cases. Syndecan-4 levels decreased in patients with CAP, especially in non-survivors. suPAR and syndecan-4 levels were positively and negatively correlated with severity scores, respectively. suPAR exhibited high accuracy in predicting SCAP among patients with CAP with an AUC of 0.835 (*p* < 0.001). In contrast, syndecan-4 exhibited poor diagnostic value for predicting SCAP (AUC 0.550, *p* = 0.187). The AUC for predicting mortality in patients with SCAP was 0.772 and 0.744 for suPAR and syndecan-4, respectively; the respective prediction threshold values were 10.22 ng/mL and 6.68 ng/mL. Addition of both suPAR and syndecan-4 to the Pneumonia Severity Index significantly improved their prognostic accuracy, with an AUC of 0.885. Regression analysis showed that suPAR ≥10.22 ng/mL and syndecan-4 ≤ 6.68 ng/mL were reliable independent markers for prediction of 30-day survival.

**Conclusion:**

suPAR exhibits high accuracy for both diagnosis and prognosis of SCAP. Syndecan-4 can reliably predict mortality in patients with SCAP. Addition of both suPAR and syndecan-4 to a clinical scoring method could improve prognostic accuracy.

**Trial registration:**

ClinicalTrials.gov, NCT03093220. Registered on 28 March 2017 (retrospectively registered).

## Background

Community-acquired pneumonia (CAP) is a very common type of respiratory infection. Despite the rapid development of new treatments, pneumonia continues to cause a high rate of complications and associated costs, and remains a major international cause of death [1, 2]. Because of the diversity of clinical conditions and the lag in a clear definition of the causative pathogen, the most challenging task for a physician is the risk stratification of patients with CAP and the subsequent administration of individual treatment [3]. CURB-65 and the Pneumonia Severity Index (PSI) are widely recommended and validated scoring methods: CURB-65 is a predictive assessment that is concisely and conveniently implemented in clinical settings [4]. PSI is a sensitive indicator in judging whether patients should be hospitalized [4]. However, both the CURB-65 and PSI scores are neither comprehensive nor exhaustive. CURB-65 assesses very few aspects of disease with low specificity, while PSI relies primarily on age and underlying diseases such that it is inaccurate in young and otherwise healthy patients. In recent years, novel biomarkers, such as soluble triggering receptor expressed on myeloid cells-1 (sTREM-1) [5], proadrenomedullin (pro-ADM) [6], and copeptin [7] have been widely validated in clinical settings. However, their sensitivity and specificity for prediction of pneumonia severity are variable and largely insufficient; thus, there is a need for new biomarkers to provide effective risk stratification and assist in clinical judgement.

Urokinase-type plasminogen activator receptor (uPAR) is a component of the plasminogen activator (PA) system. This system plays an important role in many physiological and pathological processes, including tissue remodelling [8], thrombosis [9], inflammation [10], and tumourigenesis [11]. The soluble form of uPAR (suPAR) can be detected in serum and other organic fluids [12]; its levels are increased in patients with HIV infection, malaria, tuberculosis, and sepsis, suggesting that it could serve as a useful prognostic biomarker [13]. This capability may be useful for prediction of the severity of CAP, but few studies have focused on suPAR levels in patients with CAP.

The syndecan proteins, a family of transmembrane heparan sulphate proteoglycans, bind to various extracellular effectors and regulate many processes, such as tissue homeostasis, inflammation, tumour invasion, and metastasis [14–16]. Syndecan-4 is the most well-known member of the family. Studies have demonstrated that levels of syndecan-4 increase in response to bacterial inflammation, and that syndecan-4 possesses an anti-inflammatory function in acute pneumonia [17]. However, data on the relationship between the expression of syndecan-4 and the severity of CAP are rare.

Considering the previous experimental data on suPAR and syndecan-4, we hypothesized that their serum levels might be correlated with the severity and prognosis of CAP. Thus, the aim of this study was to clarify the precise roles of suPAR and syndecan-4 in CAP, and to validate the effectiveness of these proteins as indicators of the severity of CAP and of the risk of death in severe CAP.

## Methods

### Study population

This prospective, observational study was conducted during the period of January 2014 through December 2016 among patients hospitalized in Peking University People’s Hospital, Tianjin Medical University General Hospital, Wuhan University People’s Hospital, and Fujian Provincial Hospital (ClinicalTrials.gov ID, NCT03093220). All patients in this study were diagnosed with CAP.

CAP was defined by the following criteria [18]: (1) a chest radiograph showing either a new patchy infiltrate, leaf or segment consolidation, ground glass opacity, or interstitial change; (2) at least one of the following signs – (a) the presence of cough, sputum production, and dyspnoea; (b) core body temperature >38.0 °C; (c) auscultatory findings of abnormal breath sounds and rales; or (d) peripheral white blood cell counts >10 × 10^9^/L or <4 × 10^9^/L; and (3) symptom onset that began in the community, rather than in a healthcare setting.

Severe CAP (SCAP) was diagnosed by the presence of at least one major criterion, or at least three minor criteria, as follows [19]. Major criteria: (1) requirement for invasive mechanical ventilation and (2) occurrence of septic shock with the need for vasopressors. Minor criteria: (1) respiratory rate ≥30 breaths/min; (2) oxygenation index (PaO_2_/FiO_2_) ≤250; (3) presence of multilobar infiltrates; (4) presence of confusion; (5) serum urea nitrogen ≥20 mg/dL; (6) white blood cell count ≤4 × 10^9^/L; (7) blood platelet count <100 × 10^9^/L; (8) core body temperature <36.0 °C; and (9) hypotension requiring aggressive fluid resuscitation.

The exclusion criteria were age <18 years, or the presence of any of the following: pregnancy, immunosuppressive condition, malignant tumour, end-stage renal or liver disease, active tuberculosis, or pulmonary cystic fibrosis.

### Sample size calculation

In this study, we set the type I error/significance level (two-sided) at α = 0.05 and the type II error at β = 0.10 to provide 90% power. The test standard deviation was *Z*_α_ = 1.96 and *Z*_β_ = 1.282. Assuming the mortality of non-SCAP and SCAP was *P*_0_ = 0.05 and *P*_1_ = 0.25, respectively [1, 19], the sample size was calculated as follows:

$$ {\displaystyle \begin{array}{l}R=\frac{P_1}{P_0};A={P}_1\left(1-{P}_0\right)+{P}_0\left(1-{P}_1\right);B=\left(R-1\right){P}_0\left(1-{P}_0\right);K=\left(A+B\right)\left( RA-B\right)-R{\left({P}_1-{P}_0\right)}^2\\ {}\mathrm{N}{'}_{\mathrm{non}\hbox{-} \mathrm{SCAP}}=\frac{{\mathrm{Z}}_{\upbeta}^2\mathrm{K}+{\mathrm{Z}}_{\upalpha}^2{\left(\mathrm{A}+\mathrm{B}\right)}^2+2{\mathrm{Z}}_{\upalpha}{\mathrm{Z}}_{\upbeta}\ \left(\mathrm{A}+\mathrm{B}\right)\sqrt{\mathrm{K}}\ }{{\left({P}_1\hbox{--} {P}_0\right)}^2\left(\mathrm{A}+\mathrm{B}\right)}=121\\ {}\mathrm{N}{'}_{\mathrm{SCAP}}=\frac{{\mathrm{N}}_{\mathrm{non}-\mathrm{SCAP}}}{\mathrm{R}}=25\end{array}} $$

The rate of ineligible inclusion was 10–30%. The primary number of patients with non-SCAP was calculated as follows:

$$ {\mathrm{N}}_{\mathrm{non}\hbox{-} \mathrm{SCAP}}=\frac{{\mathrm{N}}_{\mathrm{non}-\mathrm{SCAP}}^{\hbox{'}}}{1-30\%}=173 $$

The primary number of patients with SCAP was calculated as follows:

$$ {\mathrm{N}}_{\mathrm{SCAP}}=\frac{{\mathrm{N}}_{\mathrm{SCAP}}^{\hbox{'}}}{1\hbox{-} 30\%}=36 $$

**Fig. 1:**
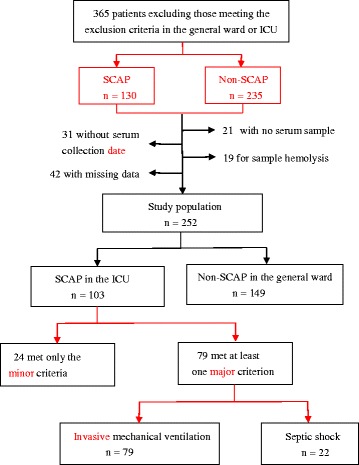
Flowchart of the study population. *SCAP* severe community-acquired pneumonia

We ultimately recruited 252 patients with CAP, including 103 with SCAP and 149 with non-SCAP. All patients with SCAP were admitted to the intensive care unit (ICU). The screening process is shown in Fig. 1. Thirty healthy people (>18 years old, without any exclusionary diseases) served as a control group. All subjects provided informed consent. This study was approved by the medical ethics committee of Peking University People’s Hospital.

### Blood sample collection

Clinical data were recorded on all patients; these included whole blood leukocyte count (WBC), blood biochemical assessment, C-reactive protein (CRP), procalcitonin (PCT), blood gas analysis, and chest images. The confusion, urea, respiratory rate, blood pressure, and age ≥65 years old (CURB-65) score [20], PSI [21], and Acute Physiology and Chronic Health Evaluation (APACHE) II [22] were calculated from clinical and laboratory data.

Peripheral venous blood samples were collected within 2 days after admission, in sterile, pro-coagulation tubes and centrifuged immediately; the resulting serum samples were stored at –80 °C until analysis.

### Measurement of suPAR and syndecan-4

Serum suPAR levels were measured using quantitative enzyme-linked immunosorbent assay (ELISA) kits (DUP00; R&D Systems, Minneapolis, MN, USA) in duplicate as instructed by the manufacturer. Briefly, test wells containing serum samples, and standard wells containing a gradient concentration of a standard protein, were analysed using a microplate assay. Absorbance at 450 nm was measured using the Multiskan FC (Thermo, Waltham, MA, USA). The detection sensitivity was 33 pg/mL and the intra-assay and inter-assay coefficients of variation were < 8%. Serum syndecan-4 levels were determined using ELISA (JP27188; Immuno-Biological Laboratories, Fujioka, Japan) in duplicate as instructed by the manufacturer. Absorbance at 570 nm was measured using the Multiskan FC. The detection sensitivity was 3.94 pg/mL and the intra-assay and inter-assay coefficients of variation were < 5%. Using the standard curve, the quantities of syndecan-4 and suPAR were calculated using CurvExpert Professional 2.6.3 (Hyams Development, Madison, WI, USA).

### Statistical analysis

Normally distributed continuous variables are expressed as means ± standard error of the mean (SEM), abnormally distributed continuous variables are expressed as median (interquartile range) and categorical variables are expressed as number (percentage). For equivalent variables with a normal distribution, the independent Student’s *t* test was utilized to compare two groups. The Mann–Whitney U test was used to compare categorical variables and abnormal distributional variables between two groups. One-way analysis of variance and the Kruskal–Wallis test were used to compare multiple groups. Correlation between variables with normal distribution was assessed using Pearson’s correlation test, while abnormal distributions were assessed using Spearman’s rho test. Receiver operating characteristic (ROC) analysis was performed to differentiate patients with CAP from those with SCAP, and to separate non-survivors from the overall patients with SCAP. Areas under the curve (AUCs), optimal threshold values, sensitivity, and specificity were calculated. Kaplan–Meier methods were used to build 30-day survival curves, and survival rates were compared using the log-rank test. Cox proportional hazards regression analyses were used to analyse the effect of an array of variables on 30-day survival.

A two-sided *p* value <0.05 was considered statistically significant; confidence intervals (CIs) were set at 95%. Statistical analyses were performed by using GraphPad Prism version 6.01 software (GraphPad Software, La Jolla, CA, USA) and MedCalc statistical software version 15.2.2 (MedCalc Software, Ostend, Belgium).

## Results

### Characteristics of the enrolled patients

From January 2014 to December 2016, 252 patients (165 male, 87 female) were enrolled and divided into two groups (103 with SCAP and 149 with non-SCAP) according to their clinical characteristics. As shown in Table 1, there were no significant differences between the SCAP and non-SCAP groups in sex, past medical history, smoking, antibiotic pre-treatment, and whether a causative pathogen was established. Chest radiographs revealed that 84.47% and 29.13% of patients with SCAP exhibited bilateral lung infection and pleural effusion, respectively; these proportions were significant higher than those observed in the non-SCAP group (29.53% and 4.70%, respectively; *p* < 0.001 for both comparisons). Laboratory analyses showed that the SCAP group exhibited a higher WBC and higher neutrophil/lymphocyte ratio (NLR) than detected in the non-SCAP group (*p* < 0.001 for both comparisons). Furthermore, serum CRP and PCT levels were substantially greater in the SCAP group than in the non-SCAP group (*p* < 0.001 for both comparisons). The CURB-65, PSI, and APACHE II scores in the SCAP group were 1 (0–2), 94.27 ± 37.42, and 15.18 ± 5.65, respectively. These scores were significantly higher than those of patients in the non-SCAP group (0 (0–1), 57.50 (35.00–72.25), 8 (6–9), respectively; *p* < 0.001 for all comparisons). The mortality rate in the SCAP group was 18.45%, while no patients with non-SCAP died in hospital.

**Table 1:** Clinical characteristics and laboratory findings of the study population

Characteristics	SCAP (*n* = 103)	Non-SCAP (*n* = 149)	*p* value
Age (years)	56.14 ± 17.32	50 (33 - 65)	0.007
Male (*n*)	73 (70.87%)	92 (61.74%)	0.135
Past medical history			
Chronic heart failure	6 (5.82%)	9 (6.04%)	0.959
Diabetes mellitus	12 (11.65%)	23 (15.44%)	0.394
Cerebrovascular disease	17 (16.50%)	13 (8.72%)	0.061
Chronic liver disease	7 (6.80%)	4 (2.68%)	0.063
COPD	7 (6.80%)	5 (3.35%)	0.208
Smoking	46 (44.66%)	76 (51.01%)	0.274
Antibiotic pre-treatment	38 (36.90%)	71 (47.65%)	0.288
Pathogen established	50 (48.54%)	65 (43.62%)	0.442
Chest x-ray			
Bilateral lung infection	87 (84.47%)	44 (29.53%)	<0.001
Pleural effusion	30 (29.13%)	7 (4.70%)	<0.001
WBC (×10^9^/L)	10.05 (6.80‒15.21)	7.49 (5.52‒10.79)	<0.001
NE%	84.00 (76.40‒90.70)	74.36 (63.95‒81.68)	<0.001
LY%	8.70 (5.05‒14.20)	16.30 (9.72‒27.05)	<0.001
NLR	10.04 (5.77‒17.99)	4.55 (2.46‒8.40)	<0.001
CRP (mg/L)	107.10 (26.00‒189.00)	46.16 (9.19‒108.7)	0.001
PCT (μg/L)	0.54 (0.20‒3.43)	0.14 (0.05‒0.64)	<0.001
CURB-65	1 (0‒ 2)	0 (0‒1)	<0.001
PSI	94 ± 37	58 (35‒72)	<0.001
APACHE II score	15 ± 6	8 (6‒9)	<0.001
Total mortality	19 (18.45%)	0 (0%)	<0.001
30-day mortality	18 (17.48)	0 (0%)	<0.001

*COPD* chronic obstructive pulmonary disease, *WBC* white blood cells, *NLR* neutrophil/lymphocyte ratio, *CRP* C-reactive protein, *PCT* procalcitonin, *CURB-65* confusion, urea, respiratory rate, blood pressure, and age ≥65 years old, *PSI* Pneumonia Severity Index, *APACHE* II Acute Physiology and Chronic Health Evaluation II

### Levels of suPAR and syndecan-4 in each group

**Fig. 2:**
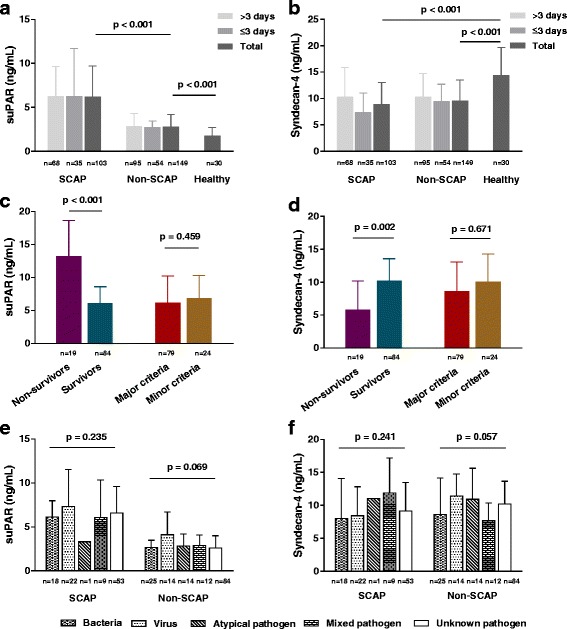
Levels of soluble urokinase-type plasminogen activator receptor (suPAR) and syndecan-4 across multiple groups. **a**, **b** Levels of suPAR and syndecan-4 in patients with severe community-acquired pneumonia SCAP, patients with non-SCAP, and healthy individuals, respectively. For suPAR, SCAP versus non-SCAP, *p* < 0.001; non-SCAP versus healthy individuals, *p* < 0.001. For syndecan-4, SCAP versus healthy individuals, *p* < 0.001; non-SCAP versus healthy individuals, *p* < 0.001. **c**, **d** Levels of suPAR and syndecan-4 in survivors and non-survivors among patients with SCAP, patients with SCAP who met at least one major criterion (major criteria), and patients with SCAP who met only minor criterion (minor criteria). For suPAR, survivors versus non-survivors, *p* < 0.001; major criteria versus minor criteria, *p* = 0.459. For Syndecan-4, survivors versus non-survivors, *p* = 0.002; major criteria versus minor criteria, *p* = 0.671. **e**, **f** Comparison of suPAR and syndecan-4 in patients with SCAP and non-SCAP for various causative pathogens; *p* > 0.05 for all comparisons

As shown in Fig. 2, serum suPAR level in healthy individuals was 1.71 ± 1.00 ng/mL, which was significantly lower than that in the non-SCAP group (2.76 (2.01–4.20) ng/mL, *p* < 0.001). The SCAP group exhibited the highest level of suPAR, 6.17 (4.37–9.72) ng/mL (compared with the non-SCAP level, *p* < 0.001). In patients with SCAP, the suPAR level of non-survivors was 13.19 (6.05–18.68) ng/mL, which was notably higher than the suPAR level of survivors (6.09 (4.01–8.63) ng/mL, *p* < 0.001).

In contrast, the expression of syndecan-4 was reduced in patients with CAP. The level of syndecan-4 in healthy individuals was 14.30 ± 5.34 ng/mL, whereas in the SCAP and non-SCAP groups the levels were 9.54 ± 5.92 and 10.15 ± 4.37 ng/mL, respectively (*p* < 0.001 for both comparisons). There was no difference in the levels of syndecan-4 between the SCAP and non-SCAP groups (*p* = 0.177, data not shown). The expression of syndecan-4 in the non-survivor SCAP group was lower than in the survivor SCAP group (5.81 ± 4.38 and 10.21 (6.20–13.56) ng/mL, respectively, *p* = 0.002).

In order to avoid possible effects of pre-admission duration of symptoms on the levels of suPAR and syndecan-4, we divided patients in two groups according to the duration of symptoms: > 3 days or ≤ 3 days. The expression of suPAR and syndecan-4 was not significantly different between the two groups both in the SCAP and the non-SCAP groups (suPAR, *p* = 0.549 (SCAP), *p* = 0.339 (non-SCAP); syndecan-4, *p* = 0.078 (SCAP), *p* = 0.635 (non-SCAP), data not shown). Moreover, the expression of suPAR and syndecan-4 was not significantly different between patients with SCAP who met at least one major criterion and those who met only minor criteria (suPAR, *p* = 0.459; syndecan-4, *p* = 0.671).

Figure 2e and f summarizes the pathogens detected in patients with CAP who were classified as having bacterial, viral, atypical pathogen (including mycoplasma pneumonia, chlamydial pneumonia, and legionella pneumonia), mixed pathogen, and unknown pathogen infections. The causative agent was detected in 50 patients with SCAP and 65 patients with non-SCAP. There were no differences in the levels of suPAR or syndecan-4 between patients with SCAP and patients with non-SCAP who exhibited different causative pathogen infections (*p* > 0.05 for all comparisons).

### Correlation between levels of suPAR and syndecan-4 and the severity of CAP

**Fig. 3:**
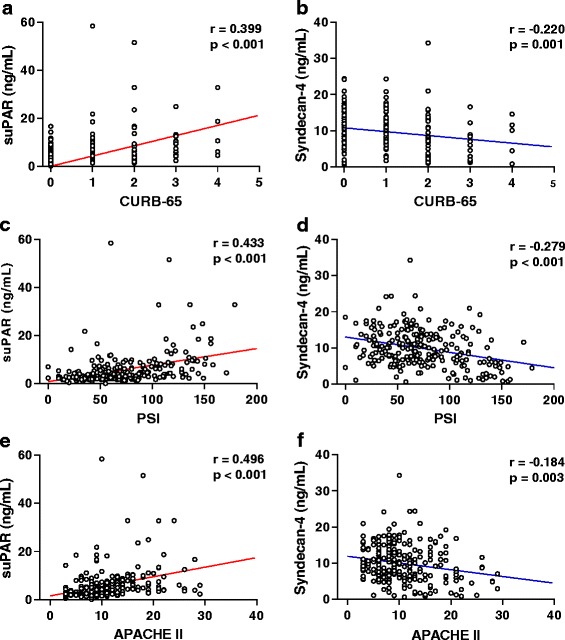
Correlation of soluble urokinase-type plasminogen activator receptor (suPAR) and syndecan-4 levels with multiple scoring systems across 252 patients with community-acquired pneumonia (CAP). *r* is the correlation coefficient. **a**, **c**, **e** Levels of suPAR were significantly positively correlated with the confusion, urea, respiratory rate, blood pressure, and age ≥65 years old (CURB-65) score (*r* = 0.399, *p* < 0.001), Pneumonia Severity Index (PSI) (*r* = 0.433, *p* < 0.001), and Acute Physiology and Chronic Health Evaluation II (APACHE II) score (*r* = 0.496, *p* < 0.001), respectively. **b, d**, **f** Levels of syndecan-4 were significantly negatively correlated with CURB-65 score (*r* = -0.220, *p* = 0.001), PSI (*r* = -0.279, *p* < 0.001), and APACHE II score (*r* = -0.184, *p* = 0.003), respectively

We chose the CURB-65, PSI, and APACHE II scoring systems to evaluate the severity of CAP. Using our entire sample of 252 patients with CAP, serum suPAR level was positively correlated with all three scoring systems: CURB-65, PSI, and APACHE II (*r* = 0.399, *r* = 0.433, and *r* = 0.496, respectively; *p* < 0.001 for all comparisons; Fig. 3). In addition, suPAR levels were also positively correlated with WBC (*r* = 0.232, *p* < 0.001), NLR (*r* = 0.351, *p* < 0.001), CRP (*r* = 0.272, *p* < 0.001), and PCT (*r* = 0.407, *p* < 0.001); suPAR levels were not positively correlated with age (*r* = 0.102, *p* = 0.107).

Conversely, syndecan-4 levels in patients with CAP were negatively correlated with all three scoring systems: CURB-65 (*r* = -0.220, *p* = 0.001), PSI (*r* = -0.279, *p* < 0.001), and APACHE II (*r* = -0.184, *p* = 0.003) (Fig. 3). Syndecan-4 levels were also negatively correlated with PCT (*r* = -0.304, *p* < 0.001), but not with WBC (*r* = 0.005, *p* = 0.943), NLR (*r* = -0.067, *p* = 0.326), CRP (*r* = -0.127, *p* = 0.081), or age (*r* = 0.023, *p* = 0.719).

### Value of suPAR and syndecan-4 in predicting SCAP in patients with CAP

**Fig. 4:**
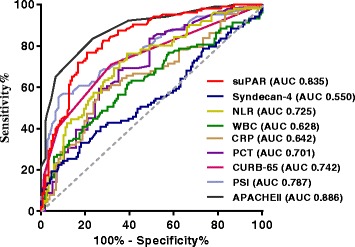
Receiver operating characteristic curve analysis of various parameters to discriminate patients with severe community-acquired pneumonia from patients with community-acquired pneumonia. *suPAR* soluble urokinase-type plasminogen activator receptor, *NLR* neutrophil/lymphocyte ratio, *WBC* whole blood leukocyte count, *CRP* C-reactive protein, *PCT* procalcitonin, *CURB-65* confusion, urea, respiratory rate, blood pressure, and age ≥65 years old score, *PSI* Pneumonia Severity Index Score, *APACHE II* Acute Physiology and Chronic Health Evaluation II score, *AUC* area under the curve

Figure 4 and Table 2 show that suPAR reliably predicted SCAP in patients with CAP, with an AUC of 0.835 (*p* < 0.001); further, suPAR prediction capability was second only to the APACHE II score (AUC 0.886, *p* < 0.001). Using a suPAR threshold value of 4.33 ng/mL for diagnosis of SCAP, the sensitivity and specificity for discriminating SCAP and CAP were 76.70% and 79.19%, respectively. Using an APACHE II score >10 as a threshold for diagnosis, the sensitivity and specificity for discriminating SCAP from CAP were 78.64% and 82.55%, respectively. Syndecan-4 was the least accurate in predicting SCAP, with an AUC of 0.550 (not statistically different, *p* = 0.187). Detailed results are shown in Fig. 4 and Table 2.

**Table 2:** Area under the curve (AUC) and thresholds for predicting SCAP in patients with CAP

	Threshold	Sensitivity (%)	Specificity (%)	AUC	*p* value	95% CI	
						Lower limit	Higher limit
suPAR (ng/mL)	>4.33	76.70%	79.19%	0.835	<0.001	0.783	0.878
Syndecan-4 (ng/mL)	≤6.10	33.01%	81.88%	0.550	0.187	0.487	0.613
WBC (×10^9^/L)	>12.15	41.75%	79.19%	0.628	0.001	0.565	0.687
NLR	>5.61	76.24%	61.02%	0.725	<0.001	0.661	0.783
CRP (mg/L)	>90.48	56.32%	73.27%	0.642	0.001	0.569	0.710
PCT (μg/L)	>0.48	53.85%	75.44%	0.701	<0.001	0.620	0.773
CURB-65	>0	70.87%	68.35%	0.742	<0.001	0.682	0.796
PSI	>87	56.86%	92.03%	0.787	<0.001	0.729	0.837
APACHE II	>10	78.64%	82.55%	0.886	<0.001	0.840	0.923

*SCAP* severe community-acquired pneumonia, *CAP* community-acquired pneumonia, *suPAR* soluble urokinase-type plasminogen activator receptor, *WBC* whole blood leukocyte count, *NLR* neutrophil/lymphocyte ratio, *CRP* C-reactive protein, *PCT* procalcitonin, *CURB-65* confusion, urea, respiratory rate, blood pressure, and age ≥65 years old score, *PSI* Pneumonia Severity Index score, *APACHE II* Acute Physiology and Chronic Health Evaluation II score

### Prognostic value of suPAR and syndecan-4 in patients with SCAP

The ability of suPAR and syndecan-4 to predict total mortality in patients with SCAP is summarized in Table 3. Notably, the AUCs for suPAR and PSI score were 0.772 and 0.787, respectively (*p* < 0.001 for both comparisons). The optimal threshold to predict death was 10.22 ng/mL of suPAR, with a sensitivity of 68.23% and specificity of 89.29%. While patients with syndecan-4 concentrations <6.68 ng/mL exhibited a noticeable increase in risk of death, this threshold yielded sensitivity and specificity of 73.68% and 71.43%, respectively for prediction of total mortality. The remaining variables, WBC, NLR, CRP, and PCT, had no prognostic value for mortality prediction in patients with SCAP (all *p* > 0.05).

**Table 3:** Area under the curve (AUC) and thresholds for predicting total mortality in patients with SCAP

	Threshold	Sensitivity (%)	Specificity (%)	AUC	*p* value	95% CI	
						Lower limit	Higher limit
suPAR (ng/mL)	≥10.22	68.23%	89.29%	0.772	<0.001	0.636	0.908
Syndecan-4 (ng/mL)	≤6.68	73.68%	71.43%	0.744	<0.001	0.618	0.870
WBC (×10^9^/L)	>16.40	36.84%	83.33%	0.558	0.489	0.393	0.723
NLR	>15.77	52.63%	71.95%	0.565	0.424	0.405	0.726
CRP (mg/L)	>164.15	42.86%	73.97%	0.533	0.701	0.363	0.703
PCT (μg/L)	>0.34	85.71%	41.56%	0.618	0.137	0.462	0.774
CURB-65	>1	68.42%	64.29%	0.723	<0.001	0.607	0.839
PSI	>113	78.95%	75.90%	0.787	<0.001	0.675	0.898
APACHE II	>14	84.21%	60.71%	0.743	<0.001	0.636	0.851

*SCAP* severe community-acquired pneumonia, *suPAR* soluble urokinase-type plasminogen activator receptor, *WBC* whole blood leukocyte count, *NLR* neutrophil/lymphocyte ratio, *CRP* C-reactive protein, *PCT* procalcitonin, *CURB-65* confusion, urea, respiratory rate, blood pressure, and age ≥65 years old score, *PSI* Pneumonia Severity Index score, *APACHE II* Acute Physiology and Chronic Health Evaluation II score

**Fig. 5:**
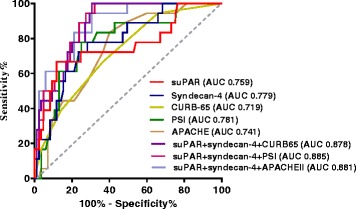
Receiver operating characteristic (ROC) curve analysis of various parameters to predict 30-day mortality in patients with SCAP

The combination of suPAR, syndecan-4, and PSI was the most accurate predictor of 30-day mortality, with an AUC of 0.885. The AUC of the combination of suPAR, syndecan-4 and CURB-65, and of suPAR, syndecan-4 and APACHE II score was 0.878 and 0.881, respectively (Fig. 5).

**Fig. 6:**
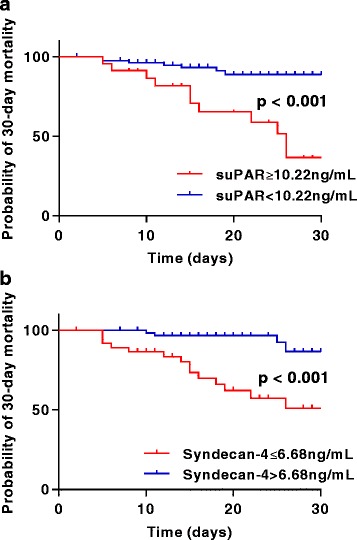
Kaplan–Meier analysis of 30-day mortality in patients with severe community-acquired pneumonia. Analysis was stratified by soluble urokinase-type plasminogen activator receptor (suPAR) (**a**) and syndecan-4 (**b**) levels

Kaplan–Meier curves were used to assess the relationship between suPAR and syndecan-4 levels in the prediction of 30-day mortality in patients with SCAP (Fig. 6). Consistent with the prediction threshold for total mortality, the optimal threshold values for 30-day mortality were also 10.22 ng/mL for suPAR (*p* < 0.001) and 6.68 ng/mL for syndecan-4 (*p* < 0.001).

In univariate Cox proportional hazards regression analysis to determine 30-day survival, suPAR levels ≥10.22 ng/mL and APACHE II scores >14 were associated with significantly higher risk ratios than any other variables. In multivariate Cox proportional hazards regression, only suPAR levels ≥10.22 ng/mL and syndecan-4 levels ≤6.68 ng/mL were strong, independent predictors of 30-day survival. Results are summarized in Table 4.

**Table 4:** Cox proportional hazards regression analysis of the effects of multiple variables on 30-day survival

Variables	Univariate analysis		Multivariate analysis	
	HR (95% CI)	*p* value	HR (95% CI)	*p* value
Age (>71 years)	3.709 (1.463‒9.405)	0.006		
WBC (>16.40×10^9^/L)	2.560 (0.956‒6.858)	0.062		
NLR (>15.77)	2.282 (0.904‒5.759)	0.081		
PCT (>0.34 μg/L)	4.069 (0.901‒18.380)	0.068		
CURB-65 (>1)	2.607 (0.977‒6.960)	0.056		
PSI (>113)	5.998 (1.967‒18.286)	0.002		
APACHE II (>14)	6.177 (1.781‒21.419)	0.004		
suPAR (≥10.22 ng/mL)	7.724 (2.887‒20.663)	<0.001	8.850 (2.716‒28.831)	<0.001
Syndecan-4 (≤6.68 ng/mL)	5.792 (1.901‒17.641)	0.002	3.976 (1.217‒12.989)	0.022

*HR* hazard ratio, *WBC* whole blood leukocyte count, *NLR* neutrophil/lymphocyte ratio, *PCT* procalcitonin, *CURB-65* confusion, urea, respiratory rate, blood pressure, and age ≥65 years old score, *PSI* Pneumonia Severity Index score, *APACHE II* Acute Physiology and Chronic Health Evaluation II score, *suPAR* soluble urokinase-type plasminogen activator receptor

## Discussion

In this prospective study of 252 patients with CAP, there were five major findings: (1) serum suPAR levels increased and syndecan-4 levels decreased in patients with CAP, especially in non-survivors, but the changes were not correlated with pre-admission duration of symptoms or the identity of the causative pathogen; (2) elevated suPAR was positively correlated with severity scores (CURB-65, PSI, and APACHE II), whereas lower syndecan-4 was negatively correlated with these same scores; (3) a suPAR threshold value of 4.33 ng/mL discriminated SCAP from CAP, with 76.70% sensitivity and 79.19% specificity, but syndecan-4 levels did not accurately predict SCAP in patients with CAP; (4) the mortality rate was significantly higher in patients with suPAR and syndecan-4 levels ≥10.22 ng/mL and ≤6.68 ng/mL, respectively, and both serum proteins independently predicted 30-day mortality; and (5) the combination of suPAR and syndecan-4 levels with the clinical severity score significantly improved the accuracy of mortality prediction. Taken together, these results suggest that serum suPAR and syndecan-4 levels can predict disease severity in patients with CAP.

suPAR has been widely studied in a variety of infectious diseases, including sepsis [23], tuberculosis [24], and HIV infection [25]. Savva et al. demonstrated that suPAR is a reliable predictor of severity of sepsis and can independently predict unfavourable outcomes in both ventilator-associated pneumonia and sepsis [26]. The findings of our study are identical to previous studies [26, 27] where suPAR levels were significantly elevated in patients with SCAP, especially in non-survivors. These results enhance our understanding of the expression of suPAR in severe infections. Furthermore, we found that suPAR levels are not correlated with specific causative agents in the same risk stratification of CAP. This is consistent with a previous study demonstrating that suPAR has no discriminatory value in patients with bacterial, viral, or parasitic infections [28].

Studies on the expression of syndecan-4 in infection are few. Nikaido et al. found that syndecan-4 levels were significantly increased in a study of 30 patients with acute mild pneumonia, and that this protein provides an anti-inflammatory function in acute pneumonia [29]. In contrast, our results show that syndecan-4 levels are significantly lower in patients with CAP compared with healthy individuals; this reduction was more pronounced in non-survivors. The lower syndecan-4 levels in our study may result from the larger sample size and the increase in CAP severity in our study, compared with the previous study. Syndecan-4 expression was elevated in mild acute pneumonia because of bacterial components that stimulated toll-like receptors 2 and 4 [29]. Importantly, the causative mechanism for lowered syndecan-4 levels in patients with CAP is not yet clear. Further, as in the analysis of suPAR, syndecan-4 levels were not statistically different among patients with CAP due to a variety of causative agents.

We investigated the correlation between suPAR and syndecan-4 levels and a variety of clinical parameters. There was strong correlation between levels of suPAR or syndecan-4 and the clinical scoring systems, suggesting that serum suPAR and syndecan-4 might aid in clinical judgment of the degree of severity. Remarkably, there was broad correlation between suPAR levels and a variety of laboratory measures: WBC, NLR, CRP, and PCT. In contrast, Wittenhagen et al. found that suPAR was not correlated with CRP in pneumococcal bacteraemia [27], but these differences may result from different patient samples. The NLR has been reported to indicate mortality and prognosis in various diseases, including tumour [30], inflammation [31], and heart failure [32]. Since NLR is convenient, easily obtained, and low cost, we included NLR as a clinical reference biomarker in this study.

The diagnostic value of suPAR is reportedly poor and was not shown to be superior to CRP or PCT. The AUC for suPAR to discriminate 197 septic ICU patients from 76 non-septic patients is 0.62 [33]. Kofoed et al. measured plasma suPAR levels in 57 patients with systemic inflammatory response syndrome (SIRS), and reported an AUC of 0.54 for suPAR (and 0.81 and 0.72 for CRP and PCT, respectively) in diagnosing bacterial infection [28]. Savva et al. reported an AUC of 0.758 for suPAR to discriminate severe sepsis or patients with septic shock within a group of 180 patients with sepsis; in the same study, the AUC for PCT was 0.652 [26]. Hoenighl et al. reported that in 132 patients with SIRS, the AUC for suPAR and PCT was 0.726 and 0.744, respectively, to differentiate patients with and without bacteraemia [34]. However, our results indicate that suPAR (AUC 0.835) had good diagnostic value in discriminating SCAP from CAP, which is comparable to APACHE II (AUC 0.886), the “gold standard” criterion for stratifying critically ill patients [22]. Taken together, these studies suggest that suPAR might have better diagnostic value in discriminating between severe and mild cases of infectious disease than in discriminating between infectious and non-infectious diseases.

Many studies focus on the prognostic value of suPAR; the AUC for suPAR in predicting in-hospital mortality ranges from 0.67 to 0.84 [35–37]. In our study, the AUC of suPAR to predict mortality was 0.772. Kaplan–Meier curves showed that suPAR ≥10.22 ng/mL was associated with significantly higher mortality risk; this threshold value is identical to at reported in prior studies [35, 38]. Further, in multivariate Cox proportional hazards regression, suPAR was an independent marker to predict 30-day mortality. This indicates that suPAR may provide a promising prognostic biomarker in SCAP.

The mechanism for increased suPAR levels in severely ill patients was uncertain. suPAR is expressed on the surface of various cells, including neutrophils, lymphocytes, macrophages, and endotheliocytes. During an inflammatory response, the presence of increased suPAR-expressing cells and the accelerated cleavage of suPAR might result in high blood levels of suPAR [35]. Furthermore, severely ill patients often present with dysfunctional blood coagulation. Excessive cytokine release activates the coagulation system in multiple ways, and contributes to the formation of a complex reaction that includes coagulation, inflammatory mediators, cytokines, and complement [39]. These processes might also promote expression of suPAR.

There has been no report on the diagnostic and prognostic value of syndecan-4. Our results show that although syndecan-4 did not discriminate SCAP from CAP, it might be used to predict mortality in patients with SCAP, with an AUC of 0.744. Further, multivariate Cox regression analysis demonstrated that the syndecan-4 level was an independent factor related to 30-day mortality. Previous studies reported that syndecan-4–deficient mice exhibit significantly higher bacterial counts, more severe pulmonary inflammation, and higher mortality, compared with wild-type mice [29, 40, 41]. Notably, these studies found that syndecan-4 expression was elevated in macrophages, endothelial cells, and epithelial cells after stimulation with lipopolysaccharide in vitro [40, 41].

Although suPAR and syndecan-4 were both good in predicting 30-day mortality, none was better than PSI. Remarkably, our results show that the addition of both suPAR and syndecan-4 to a clinical severity scoring method significantly improved their prognostic accuracy. The severity score alone is often insufficient to obtain satisfactory predictive accuracy. Therefore, biomarkers are thought to be able to better stratify patients. Mid-region proadrenomedullin (MR-proADM) has been hitherto the best single predictor of short-term and long-term mortality. Notably, the AUC of a combination of MR-proADM and PSI for 30-day mortality prediction can reach 0.914 [6].

Our study has certain limitations. Only serum suPAR and syndecan-4 levels were detected at the time of admission; dynamic and follow-up changes (in response to treatment) were not investigated. Besides, suPAR is a non-specific inflammatory marker that has been shown to be increased in diabetes mellitus [42], liver disease [43], and heart failure [44]. While our study enrolled a number of patients with those comorbidities (as shown in Table 1), the pre-admission levels of suPAR should be tested. The effects of changes in suPAR and syndecan-4 expression during the pathogenesis of CAP should be further investigated.

## Conclusions

In conclusion, we demonstrated that serum suPAR is elevated, and serum syndecan-4 is reduced, in patients with SCAP. suPAR is able to accurately predict SCAP and mortality in patients with CAP. Further, we revealed that syndecan-4 has no diagnostic value but that it can serve as a prognostic biomarker in patients with SCAP. The combination of both suPAR and syndecan-4 with clinical scores significantly improved their 30-day mortality prediction.

## Abbreviations

APACHE II: Acute Physiology and Chronic Health Evaluation II
AUC: Area under the curve
CAP: Community-acquired pneumonia
CI: Confidence interval
CRP: C-reactive protein
CURB-65: Confusion, urea, respiratory rate, blood pressure, and age ≥65 years old, scoring system
MR-proADM: Mid-region proadrenomedullin
NLR: Neutrophil/lymphocyte ratio
PA: Plasminogen activator
PCT: Procalcitonin
pro-ADM: Proadrenomedullin
PSI: Pneumonia Severity Index
ROC: Receiver operating characteristic
SCAP: Severe community-acquired pneumonia
suPAR: Soluble urokinase-type plasminogen activator receptor
uPAR: Urokinase-type plasminogen activator receptor
WBC: Whole blood leukocyte count
